# Supplementary material for: Trimethylamine-N-Oxide Postprandial Response in Plasma and Urine Is Lower After Fermented Compared to Non-Fermented Dairy Consumption in Healthy Adults
Source: Nutrients. 2020 Jan 16;12(1):234. doi: 10.3390/nu12010234 (PMC7019705; doi:10.3390/nu12010234)
Supplement: Supplementary file 1 [file nutrients-12-00234-s001.pdf]

nutrients

SUPPLEMENTAL FIGURES

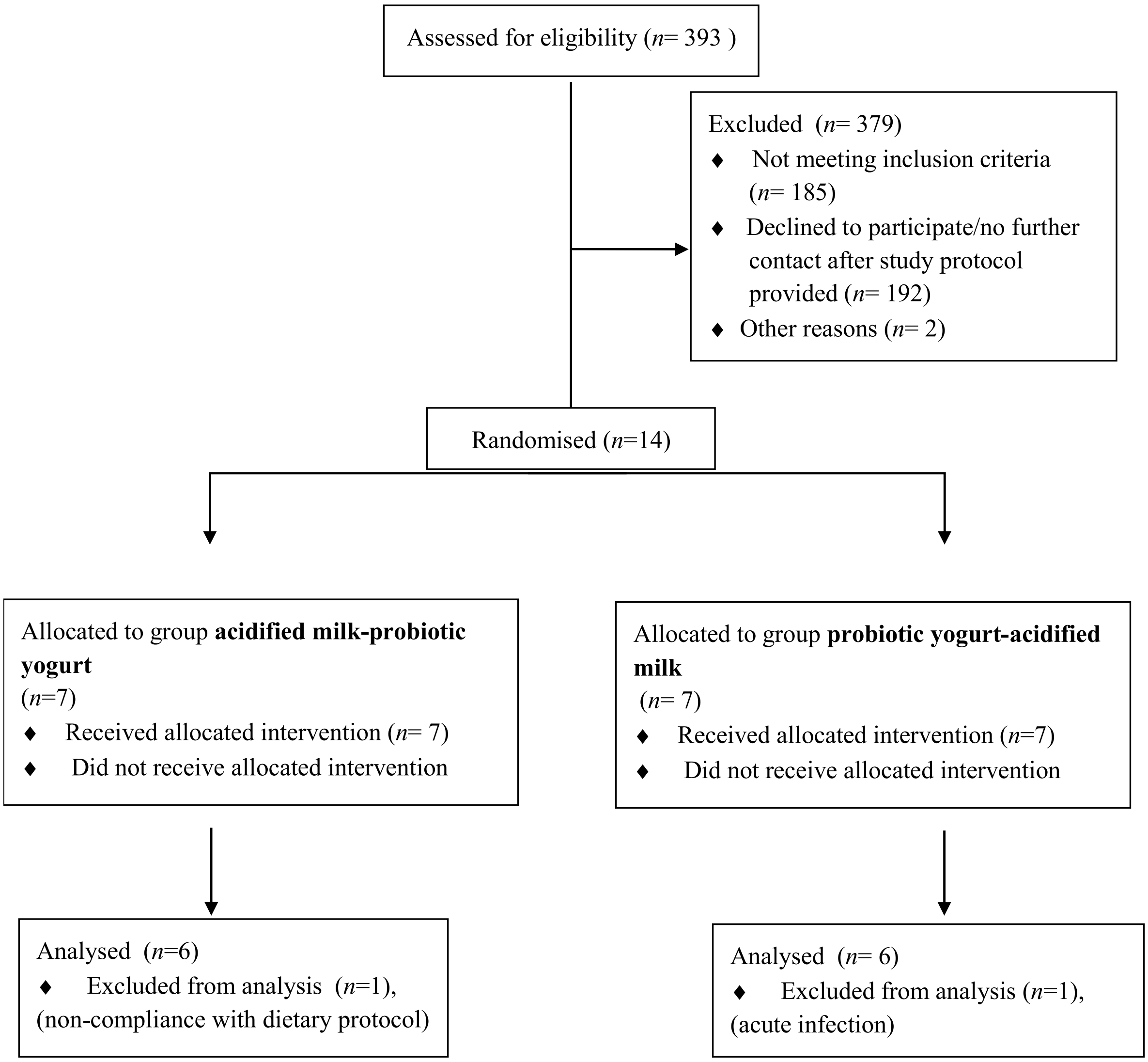

Figure S1. Flow chart of the F3 study. Adapted from CONSORT 2010 (reproduced from Burton *et al.*, with permission from authors[1]).

[1] Burton, K.J.; Rosikiewicz, M.; Pimentel, G.; Butikofer, U.; von Ah, U.; Voirol, M.J.; Croxatto, A.; Aeby, S.; Drai, J.; McTernan, P.G., et al. Probiotic yogurt and acidified milk similarly reduce postprandial inflammation and both alter the gut microbiota of healthy, young men. *Br J Nutr* **2017**, 10.1017/S0007114517000885, 1-11, doi:10.1017/S0007114517000885.

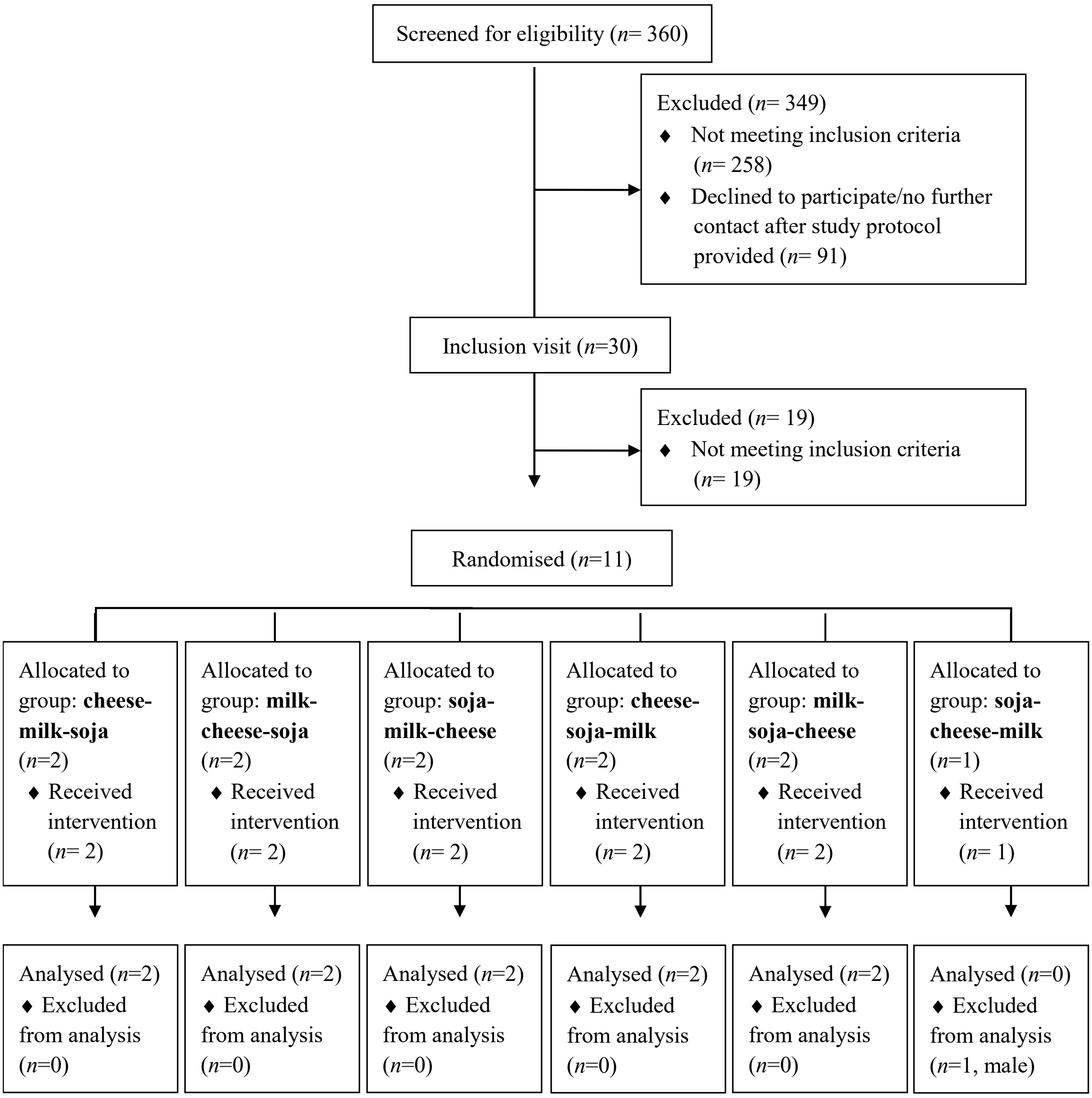

Figure S2. Flow chart of the FoodBAll dairy study. Adapted from CONSORT 2010.

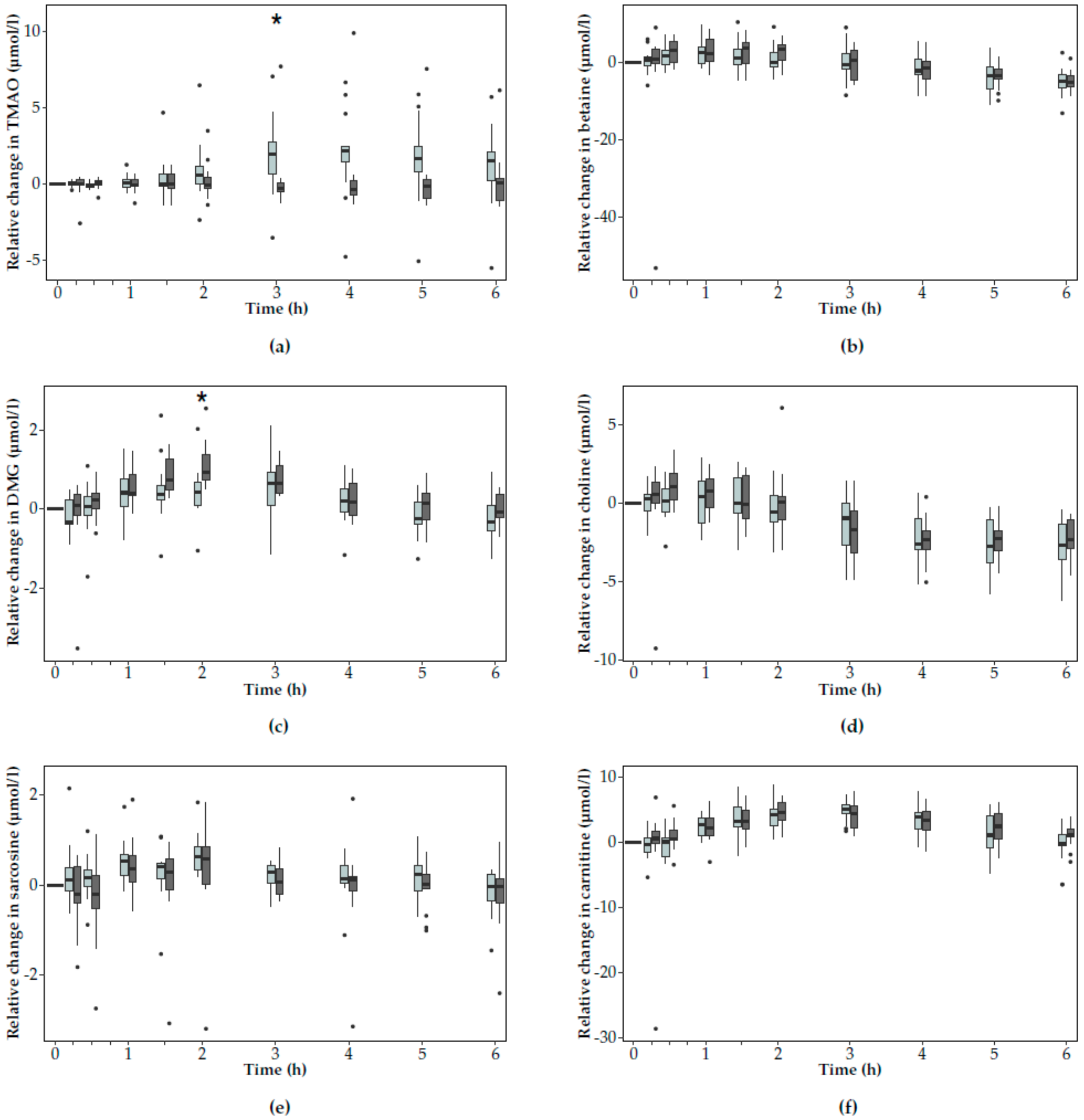

Figure S3. Postprandial changes in concentrations of TMAO and TMAO-related metabolites in plasma after the consumption of fermented and non-fermented dairy products (study 1). The postprandial changes (relative to fasting values) in metabolites concentrations (a–f) after acidified milk (light grey) and yogurt (dark grey) consumption are compared at each time point using paired Wilcoxon signed-rank test (significance at $P < 0.05$, indicated by *). Plots show the IQR (box), the median dividing the IQR (—), with whiskers that extend 1.5 x IQR beyond the IQR and outliers outside this range identified (•).

DMG, N,N-dimethylglycine; TMAO, trimethylamine-*N*-oxide.

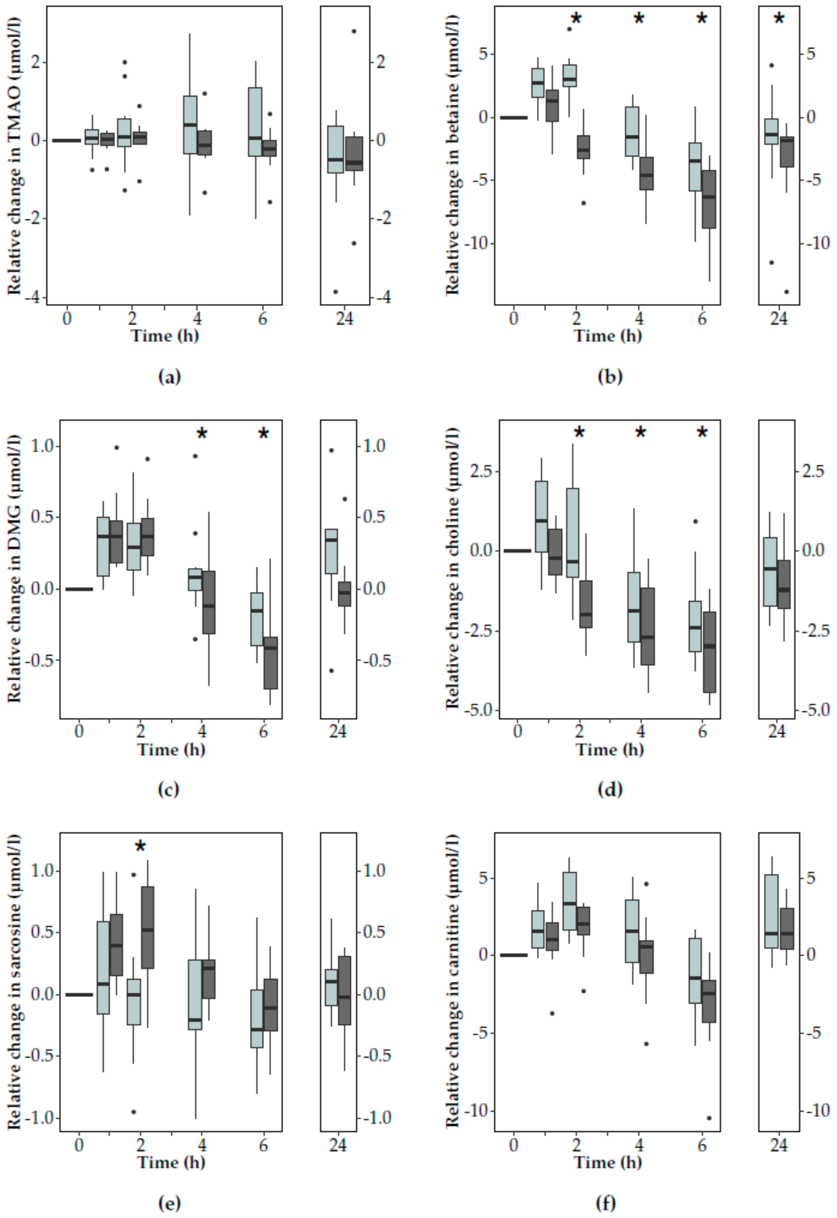

Figure S4. Postprandial changes in concentrations of TMAO and TMAO-related metabolites in plasma after the consumption of fermented and non-fermented dairy products (study 2). The postprandial changes (relative to fasting values) in metabolite concentrations (a–f) after milk (light grey) and cheese (dark grey) consumption are compared at each time point using paired Wilcoxon signed-rank test (significance at $P < 0.05$, indicated by *). Plots show the IQR (box), the median dividing the IQR (—), with whiskers that extend 1.5 x IQR beyond the IQR and outliers outside this range identified (•).

DMG, N,N-dimethylglycine; TMAO, trimethylamine-*N*-oxide.

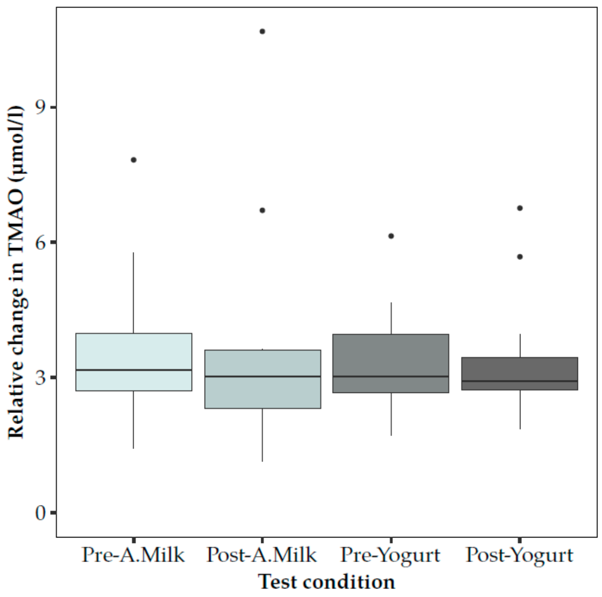

Figure S5. Boxplots illustrating the fasting concentrations of TMAO in plasma before (pre-) and after (post-) two-week bi-daily consumption of fermented and non-fermented dairy products (study 1). The relative changes in fasting concentration of TMAO after acidified milk (light grey) and c (dark grey) consumption are compared using paired Wilcoxon signed-rank test ($P < 0.05$). No significant differences are observed. Plots show the IQR (box), the median dividing the IQR (—), with whiskers that extend 1.5 x IQR beyond the IQR and outliers outside this range identified (•).

DMG, N,N-dimethylglycine; TMAO, trimethylamine-*N*-oxide.

TABLES

	**PLASMA**	
	**Food**	**TMAO iAUC IQR (Q3-Q1) (A-U.)**
**STUDY 1**	Acidified milk	5.26
	Yogurt	3.94
**STUDY 2**	Milk	6.41
	Cheese	1.60
	**URINE**	
	**Food**	**TMAO IQR (Q3-Q1) 6 h pool (μM/Cr (mg/dL))**
**STUDY 1**	Acidified milk	1.48
	Yogurt	1.00
**STUDY 2**	Milk	71.3
	Cheese	17.9

Table S1. Summary of the variation (indicated by IQR) in 6 h postprandial TMAO responses assessed in plasma (iAUC) and urine (6 h pool) after consumption of fermented (yogurt and cheese) and non-fermented (acidified milk and milk) dairy foods.

Cr, creatinine; iAUC, incremental AUC; TMAO, trimethylamine-*N*-oxide.

**Metabolite**	**Median net iAUC after acidified milk (IQR) (A.U.)**	**Median net iAUC after yogurt (IQR) (A.U.)**	***P* value**
TMAO	5.63 (4.43 - 9.68)	17.1 (13.5 - 21.0)	0.04*
Betaine	-2.42 (-24.1 - 7.69)	-0.41 (-7.66 - 9.51)	0.89
DMG	1.18 (0.42 - 2.11)	-7.99 (-10.8 - -2.80)	0.17
Choline	-8.88 (-14.1 - -0.74)	2.72 (1.11 - 4.04)	1.00
Sarcosine	1.18 (1.02 - 2.10)	1.11 (-1.15 - 2.21)	0.34
Carnitine	17.6 (5.61 - 23.4)	-1.00 (-2.81 - 1.14)	0.64

Table S2**.** Comparison of postprandial responses in plasma (represented by the net iAUC) to a single consumption of acidified milk or yogurt in study 1. Evaluation of difference by paired Wilcoxon signed-rank test (* $P < 0.05$).

A.U., arbitrary units; DMG, N,N-dimethylglycine; iAUC, incremental AUC; TMAO, trimethylamine-*N*-oxide.

Metabolite	Median net iAUC after milk (IQR) (A.U.)	Median net iAUC after Cheese (IQR) (A.U.)	*P* value
TMAO	2.09 (-1.34 - 5.07)	-0.27 (-0.85 - 0.75)	0.10
Betaine	0.44 (-6.29 - 7.75)	-21.8 (-24.7 - -13.8)	0.006*
DMG	0.77 (0.41 - 1.28)	0.24 (-0.54 - 1.02)	0.19
Choline	-6.48 (-9.16 - 3.04)	-11.4 (-15.9 - -5.34)	0.03*
Sarcosine	-0.78 (-1.39 - 0.91)	1.79 (0.47 - 2.46)	0.06
Carnitine	8.34 (0.32 - 19.1)	4.12 (-2.97 - 5.84)	0.38

Table S3. Comparison of postprandial responses in plasma (represented by the net iAUC) to a single consumption of milk or cheese in study 2. Evaluation of difference by paired Wilcoxon signed-rank test (* $P < 0.05$).

A.U., arbitrary units; DMG, N,N-dimethylglycine; iAUC, incremental AUC; TMAO, trimethylamine-*N*-oxide.

Metabolite	Median 6 h pool acidified milk (IQR) (μM/Cr (mg/dL))	Median 6 h pool yogurt (IQR)) (μM/Cr (mg/dL))	*P* value
TMAO	4.62 (4.06 - 5.54)	2.85 (2.59 - 3.59)	0.013*
Betaine	0.42 (0.31 - 0.50)	0.56 (0.40 - 0.91)	0.005*
DMG	0.32 (0.23 - 0.41)	0.51 (0.30 - 0.54)	0.001*
Choline	0.17 (0.15 - 0.21)	0.17 (0.15 - 0.23)	1.00
Sarcosine	0.01 (0.00 - 0.02)	0.01 (0.01 - 0.02)	0.86
Carnitine	0.54 (0.29 - 1.08)	0.80 (0.21 - 1.29)	0.27

Table S4. Comparison of 6 h postprandial urine samples after a single consumption of acidified milk or yogurt in study 1. Evaluation of differences by paired Wilcoxon signed-rank test (* $P < 0.05$).

Cr, creatinine; DMG, N,N-dimethylglycine; iAUC, incremental AUC; TMAO, trimethylamine-*N*-oxide.
